# *O-*Linked N-Acetylglucosamine Modification of Mitochondrial Antiviral Signaling Protein Regulates Antiviral Signaling by Modulating Its Activity

**DOI:** 10.3389/fimmu.2020.589259

**Published:** 2021-02-02

**Authors:** Junghwa Seo, Yun Soo Park, Tae Hyun Kweon, Jingu Kang, Seongjin Son, Han Byeol Kim, Yu Ri Seo, Min Jueng Kang, Eugene C. Yi, Yong-ho Lee, Jin-Hong Kim, Boyoun Park, Won Ho Yang, Jin Won Cho

**Affiliations:** ^1^Glycosylation Network Research Center, Yonsei University, Seoul, South Korea; ^2^Interdisciplinary Program of Integrated OMICS for Biomedical Science, Graduate School, Yonsei University, Seoul, South Korea; ^3^Department of Molecular Medicine and Biopharmaceutical Sciences, School of Convergence Science and Technology and College of Medicine or College of Pharmacy, Seoul National University, Seoul, South Korea; ^4^Department of Internal Medicine, Yonsei University College of Medicine, Seoul, South Korea; ^5^Department of Biological Sciences, College of Natural Sciences, Seoul National University, Seoul, South Korea; ^6^Department of Systems Biology, College of Life Science and Biotechnology, Yonsei University, Seoul, South Korea

**Keywords:** host defense mechanism, innate immunity, mitochondrial antiviral signaling protein, *O*-linked N-Acetylglucosamine (*O*-GlcNAc), RIG-I-like receptors signaling

## Abstract

Post-translational modifications, including *O*-GlcNAcylation, play fundamental roles in modulating cellular events, including transcription, signal transduction, and immune signaling. Several molecular targets of *O*-GlcNAcylation associated with pathogen-induced innate immune responses have been identified; however, the direct regulatory mechanisms linking *O*-GlcNAcylation with antiviral RIG-I-like receptor signaling are not fully understood. In this study, we found that cellular levels of *O*-GlcNAcylation decline in response to infection with Sendai virus. We identified a heavily *O*-GlcNAcylated serine-rich region between amino acids 249–257 of the mitochondrial antiviral signaling protein (MAVS); modification at this site disrupts MAVS aggregation and prevents MAVS-mediated activation and signaling. *O*-GlcNAcylation of the serine-rich region of MAVS also suppresses its interaction with TRAF3; this prevents IRF3 activation and production of interferon-β. Taken together, these results suggest that *O*-GlcNAcylation of MAVS may be a master regulatory event that promotes host defense against RNA viruses.

## Introduction

Innate immune signaling in vertebrate species is orchestrated by three types of receptor families; toll-like-receptors (TLRs), NOD-like receptors, and RIG-I-like receptors (RLRs). Specifically, when an RNA virus enters a host cell, the viral double-stranded (ds)RNA exposed to the cytoplasm is detected by RLRs, as opposed to that recognized by TLRs on the cell surface. Upon detection of the viral dsRNA, RIG-I undergoes various post-translational modifications, including acetylation and ubiquitination (1–3). This activated form of RIG-I then binds to mitochondrial antiviral signaling protein (MAVS), which is located in the mitochondrial outer membrane (4). The RIG-I-MAVS complex induces the formation of fully activated prion-like aggregates via caspase recruitment domain (CARD-CARD) interactions (5, 6). The resulting MAVS aggregates then recruit the E3 ligases, TRAF2, 3, and 6, to promote K63-linked ubiquitination to activate downstream kinases, including TBK1 and IKKϵ (3, 7). Interferon (IFN) regulatory factor-3 (IRF3) and NF-κB are then phosphorylated sequentially and translocated to the nucleus; these transcription factors (TFs) promote the production of type I IFNs, pro-inflammatory cytokines, and antiviral factors that are secreted and can modulate the responses of neighboring cells (5). This process ultimately contributes to host defense by promoting virus clearance.

Immune cells have well-established TLR systems that facilitate rapid and precise responses to pathogens. Macrophages play a significant role in governing optimal interactions between the TLRs and RLR. By contrast, expression of TLR genes in both epithelial cells and fibroblasts is remarkably low (8). As such, MAVS-mediated RLR signaling is a critical component of the immunological response to RNA virus infection of epithelial cells. Macrophages rely on important connections between glucose metabolism and antiviral host responses (9, 10). Specifically, increased glucose metabolism in macrophages at relatively early time points after virus infection can accelerate of the development of the innate immune response (9, 10). However, the relationship between glucose metabolism and RLR signaling in epithelial cells at later time point in infection, notably in cells that with ubiquitous expression of MAVS, should be further evaluated.

The processes leading to *O-*linked N-Acetylglucosamine (*O*-GlcNAc) are regulated by various cellular signals and external stress stimuli and is among the most sensitive and dynamic of the post-translational modifications (11, 12). Many proteins in the nucleus, cytoplasm, and mitochondria are targets of *O*-GlcNAc transferase (OGT) (13). OGT catalyzes the reversible attachment of a GlcNAc moiety to the hydroxyl groups of the serine or threonine residues using UDP-GlcNAc from the hexosamine biosynthetic pathway (HBP) as a substrate; *O*-GlcNAcase (OGA) catalyzes the removal of *O*-GlcNAc from the protein target (11–14). *O*-GlcNAc modifications serve to modulate mitochondrial motility through factors including Milton 1 (15) and mitochondrial trafficking via the actions of trafficking kinesin (TRAK) protein (16); likewise, *O*-GlcNAc modifications of proteins including Drp1 and OPA1 serve to regulate mitochondrial fission and fusion (17). *O*-GlcNAcylation has been identified at the C-terminus of MAVS, which is a key adapter protein in the RLR signaling pathway (9, 10); this modification has been shown to promote antiviral immunity. Interestingly, the serine/threonine content of MAVS is ~20% (i.e.,108 of 540 amino acids); as such, many sites are candidates for post-translational modification besides those previously identified.

Pathogen infection threatens host cell survival. Results from several reports have suggested a link between pathogen infection and levels of intracellular *O*-GlcNAcylation. For example, the DNA viruses herpes simplex virus (HSV) and the cytomegalovirus rely on OGT activity and *O*-GlcNAcylation to support their replication, proliferation, and propagation (18). Furthermore, high levels of OGT activity and *O*-GlcNAcylation were detected in human papillomavirus (HPV)-induced cervical neoplasms; increased levels of *O*-GlcNAcylation were observed in mouse embryonic fibroblasts in response to HPV E6 oncoprotein overexpression (19). Despite these findings, the way in which host cells defend themselves from virus infection and promote innate immune signaling via *O*-GlcNAcylation is still unclear.

Here, we report that transcription of OGT is dramatically decreased at early time points after infection with an RNA virus; this contributes to the depletion of the *O*-GlcNAcylation of MAVS at later time points after infection, which serves to promote an innate immune response. We also identified a heavily *O*-GlcNAcylated serine-rich region of MAVS that spans amino acids Serine 249 through Serine 257. *O*-GlcNAcylation was blocked via deletion of the MAVS serine-rich region (amino acids 249–257); this promoted the aggregation of MAVS, strengthened the interaction between MAVS and TRAF3, and ultimately resulted in an increase in the production of interferon-β (IFN-β). Collectively, these results provide new insights into the cross-talk between host defense mechanisms and *O*-GlcNAc metabolism and reveal both novel and distinctive antiviral functions mediated by *O*-GlcNAcylation.

## Materials and Methods

### Cell Cultures

All epithelial cell lines used in this study were incubated at 37°C in 5% CO_2_. HEK293 and MDA-MB-231 cells were cultured in Dulbecco’s Modified Eagle’s Medium (DMEM, Lonza, Basel, Switzerland) supplemented with 10% fetal bovine serum (HyClone, Logan, UT, USA). A549 was cultured in ATCC-formulated F-12K Medium (ATCC, USA, Catalog No. 30-2004) supplemented with 10% fetal bovine serum. HT-29, and U937 were cultured in Roswell Park Memorial Institute (RPMI) 1640 medium with HEPES and L-glutamine (HyClone, South Logan, Utah, USA, SH30255.01).

### Sendai Virus Infection

SeV (Cantell strain; Charles River Laboratories) was used to infect cells at a concentration of 100 hemagglutination units (HAU)/ml. HEK293, A549, and other cells were plated 24 h before infection. Cells were infected with SeV in serum-free DMEM or RPMI-1640 for 1 h at 37°C, washed with 1× phosphate-buffered saline, and incubated in complete medium for various periods of time as indicated for each experiment.

### Plasmids and Transfection

The plasmid pCS4-Myc-MAVS was kindly provided by Dr. Yukiko Gotoh (University of Tokyo, Tokyo, Japan). Myc-MAVS-1-265 and Myc-MAVS-266-540 were cloned into the pcDNA3.1-MycHisC expression vector. Wild-type and mutant forms of human MAVS wild type were prepared by PCR and cloned into the pRK5-Flag expression vector (Genentech). The mutations were confirmed by DNA sequence analysis (Bionics, South Korea). The plasmid pEFBos-Flag-N-RIG was kindly provided by Dr. Michael Gale (University of Washington School of Medicine, Washington, USA), and the plasmid pKH3-3XHA-TRAF3 was kindly provided by Dr. Ying Zhu (Wuhan University, Wuhan, China). Human Flag-OGT, Flag-OGA, and Myc-OGT were cloned into the p3XFlag-CMV™-7.1 expression vector (Sigma-Aldrich) and into the pcDNA3.1-MycHisC vector. For transient overexpression, cells were transfected using Omicsfect (OmicsBio, Taipei, Taiwan) in serum-free medium according to the manufacturer’s instructions for 24–48 h. In this paper, we transfected cells with DNA expression vectors or with the empty vector as control.

### Reagent and RNAi Interference

Cells were treated with 1 μM Thiamet-G (Sigma-Aldrich) for 2 to 4 h before SeV infection or transfections. Cycloheximide (Sigma-Aldrich) treatment was performed for the times indicated. Cells were collected at each time point indicated. Transfection with poly (I:C) (Sigma-Aldrich) was performed using the TransIT-2020 Transfection Reagent (Mirus Bio, USA) according to the manufacturer’s instructions. To establish knock-down of OGT or MAVS via siRNA interference, cells were transfected with siRNAs targeting OGT or MAVS with Lipofectamine RNAi MAX (Invitrogen, USA) according to the manufacturer’s transfection protocol. The siRNA sequences targeting these proteins were as follows:

siCTL (control), #1, duplex, Cat.SN-1002, Bioneer, Korea siOGT, #1, sense, 5′- UAAUCAUUUCAAUAACUGCUUCUGC (dTdT) - 3′ siOGT, #1, antisense, 5′- GCAGAAGCAGUUAUUGAAAUGAUUA (dTdT) - 3′ siMAVS, #1, sense, 5′- GAGUCAGCCAUGAUUGCUU (dTdT) - 3′ siMAVS, #1, antisense, 5′- AAGCAAUCAUGGCUGACUC (dTdT) - 3′ siMAVS, #2, sense, 5′- GCUCACCAAUCCAGCACCA (dTdT) - 3′ siMAVS, #2, antisense, 5′- UGGUGCUGGAUUGGUGAGC (dTdT) - 3′.

### Western Blotting, Succinylated-Wheat Germ Agglutinin Lectin Precipitation, and IP

For Western blotting assays, cells were lysed with buffer A (150 mM NaCl, 1 mM EDTA, 50 mM Tris-HCl (pH7.4), 1% NP-40) supplemented with a protease inhibitor cocktail (Roche, Mannheim, Germany) and a phosphatase inhibitor cocktail (Roche, Germany). Preparation of sWGA was as previously described (20). Briefly, 1.5 to 2 mg of total cell lysates were incubated with agarose-conjugated sWGA (Vector Laboratories, Burlingame, CA, USA) overnight at 4°C. For IP, 1.5 to 2 mg of total cell lysates were incubated with agarose-conjugated anti-FLAG antibody (MBL, Woburn, USA) or anti-Myc antibody (MBL, Woburn, USA) for 2 h at 4°C. For IP of endogenous MAVS, 3 mg of total cell lysates were incubated with anti-MAVS antibody (#166583, Santa Cruz, Dallas, TX, USA) overnight at 4°C followed by agarose-conjugated protein A/G (Santa Cruz, Dallas, TX, USA) for 2 h at room temperature. Purified proteins in sWGA/IP precipitates were washed four times with buffer B (150 mM NaCl, 2 mM EGTA, 2 mM MgCl_2_, 20 mM HEPES (pH 7.4), and 0.1% NP-40) and were eluted with sodium-dodecyl sulfate (SDS) loading buffer at 95°C for 5 min. The eluents were analyzed via Western blots probed with specific antibodies described in the section to follow. For co-IP experiments, 1.5 to 2 mg of total cell lysates were lysed with buffer C (150 mM NaCl, 0.1 mM EDTA, 1 mM dithiothreitol, 50 mM Tris-HCl (pH 7.4), and 0.5% Triton-X100). For Western blotting, 20 to 30 μg of total cell lysate was loaded onto 8% to 10% SDS-PAGE gel. Exceptionally, 60 μg of total cell lysate was loaded on the SDS-PAGE gel to detect p-IRF3. The same amount of protein was loaded in each experiment. After separation onto SDS-PAGE gel, proteins are transferred to NC membrane to detect signals. EZ-Western kit (DoGenBio) or SuperSignal West Femto Chemiluminescent Substrate (Thermo Fisher Scientific, Inc.) and Amersham Imager 600 (GE Healthcare Life Sciences, Little Chalfont, UK) were used to signal detection. For quantifying signals, the immunoreactive protein band was detected and the integrated signal intensity was measured using AI600 imager system software. Thereafter, *O*-GlcNAcylation levels were normalized to integrated signal intensity of β-actin or GAPDH, the loading control of the same gel for each cell type. OGT protein levels were normalized to β-actin, a loading control of the same gel. To quantify proteins and *O*-GlcNAcylation levels, Loading control proteins including β-Actin and GAPDH, and target proteins to observe or *O*-GlcNAc levels were measured using AI600 imager system (GE Healthcare, Chicago, IL, USA) software.

### Antibodies

The primary antibodies used for Western blotting included anti-*O*-GlcNAc (#MA1-072, Thermo Scientific), anti-OGT (DM17, #O6264, Sigma-Aldrich), anti-OGA (EPR7154(B) #ab124807, Abcam), anti-MAVS (#A300-782A, Bethyl Laboratories), anti-c-Myc (#sc-789, Santa Cruz), anti-GAPDH (#sc-32233, Santa Cruz), anti-IRF3 (#sc-8092, Santa Cruz), anti-SeV (#PD029, MBL), anti-FLAG (#PM020, MBL), anti-β-actin (#4970, CST), and anti-p-IRF3 (D601M, #29047, CST). NC membranes were probed with primary antibodies followed by secondary antibodies conjugated with HRP(horseradish peroxidase) in a ratio of 1:10000. Secondary antibodies included goat anti-rabbit IgG (#111-035-003, Jackson Immunoresearch), goat anti-mouse IgG (#115-035-003, Jackson Immunoresearch), and goat anti-mouse IgM (#115-005-020, Jackson Immunoresearch).

### Semi-Denaturing Detergent-Polyacrylamide Gel Electrophoresis, Isolation of Mitochondria, and Semi-Denaturing Detergent-Agarose Gel Electrophoresis

SDD-PAGE was performed as previously described (21) to detect SDS-resistant high-molecular weight MAVS aggregates. Briefly, HEK293 cells were transfected with plasmids encoding Flag-MAVS or Myc-OGA or empty vector alone; after 24 h, cells were harvested and lysed with Buffer A. The lysates were mixed with a 4× sample buffer both with and without β-mercaptoethanol followed by SDD-PAGE. To detect endogenous MAVS aggregates, mitochondria were isolated from cultured cells using a Mitochondria Isolation Kit (#89874, Thermo Scientific, Rockford, IL, USA) according to the manufacturer’s instructions. Then, the mitochondrial fraction was re-suspended in 1% diaminodiphenylmethane-containing lysis buffer and analyzed by 2% agarose SDD-AGE.

### Real-Time Quantitative RT-PCR

Total RNA was extracted from cultured cells using the TRIzol reagent (Invitrogen, USA). To obtain cDNA, RT was performed on 1 μg of the extracted total RNA using ReverTra Ace qPCR RT Master Mix (Toyobo, Japan) according to the manufacturer’s instructions. The qPCR was conducted using SYBR Premix Ex Taq (Takara, Japan) using a CFX96™ real-time system (Bio-Rad). Relative mRNA levels of OGT and IFN-β were normalized to those of actin. The qPCR primer sequences were as follows:

IFN-β, Forward 5′-AAA CTC ATG AGC AGT CTG CA-3′

IFN-β, Reverse 5′-AGG AGA TCT TCA GTT TCG GAG G-3′

OGT, Forward 5′-CTT TAG CAC TCT GGC AAT TAA ACA G-3′

OGT, Reverse 5′-TCA AAT AAC ATG CCT TGG CTT C-3′

Actin, Forward 5′-AGA GCT ACG AGC TGC CTG AC-3′

Actin, Reverse 5′-AGC ACT GTG TTG GCG TAC AG-3′

### Mapping O-GlcNAc Sites in Mitochondrial Antiviral Signaling Protein

The extracted peptides were dissolved in Solvent A (0.1% formic acid in H_2_O). Peptides were separated using a PepMap™ RSLC C18 column (Thermo Fisher Scientific, San Jose, CA) with a linear gradient of 2% to 38% Solvent B (0.1% formic acid in acetonitrile) over 75 min at a flow rate of 300 ml/min. The sample was analyzed by Orbitrap Fusion Lumos Tribrid mass spectrometer (Thermo Fisher Scientific, San Jose, CA) that interfaced with an Easy nanoLC 1200 system (Thermo Fisher Scientific, San Jose, CA). The spray voltage was set to 1.9 kV and the temperature of the heated capillary was set to 275°C. The equipment was set in data-dependent mode with one survey MS scan followed by 10 MS/MS scans and a dynamic exclusion time of 30 s. Full scans were acquired at 350 to 1600 m/z. The resolution on mass spectrometry was 120,000 and the automatic gain control (AGC) target was set to 4e^5^. The MS/MS scans had a resolution of 30,000; the AGC target was set to 5e^4^ for high-energy collisional dissociation fragmentation. If oxonium product ions (m/z 204.0867, 138.0545) were observed in the higher-energy collisional dissociation (HCD) spectra, EThcD with user-defined charge dependent reaction time (45.20 ms for 3+ charged, 25.42 ms for 4+ charged) with 15% or 17% HCD supplemental activation was performed in a subsequent scan on the same precursor ion selected for HCD. The EThcD MS/MS scans had a resolution of 30,000 with the AGC target set to 1e5. The maximum injection time was 120 ms.

Collected MS/MS data were used in a search of the decoy UniProt human database (Release 2019_11, 186 532 entries) for the estimation of the false discovery rate (FDR) with the Sequest HT software in Proteome Discoverer 2.2 (Thermo Fisher Scientific). Precursor and fragment ion tolerance were set to 10 ppm and 0.02 Da, respectively. Trypsin was chosen as the enzyme with a maximum allowance of up to two missed cleavages. The following modifications were defined as static modification of carbamidomethyl (Cys), dynamic modification of HexNAc 203.079 Da (Ser and Thr), Phospho 79.966 Da (Ser and Thr), and Oxidation (Met). The data were also searched against the decoy database and the results were used to calculate q values of peptide–spectrum matches (PSMs) using the Fixed Value PSM Validator within the Proteome Discoverer. Peptide and protein results were filtered to a 1% FDR.

### Statistical Analysis

Data are presented as mean ± SEM based on at least three independent experiments. Statistical analysis was performed using two-tailed Student t-tests to compare results from two groups and by One-way analysis of variance for multiple groups. A P values of <0.05 considered as significant.

## Results

### Reduced *O*-GlcNAcylation in Response to Sendai Virus Infection Stimulates the Innate Immune Response

**Figure 1:**
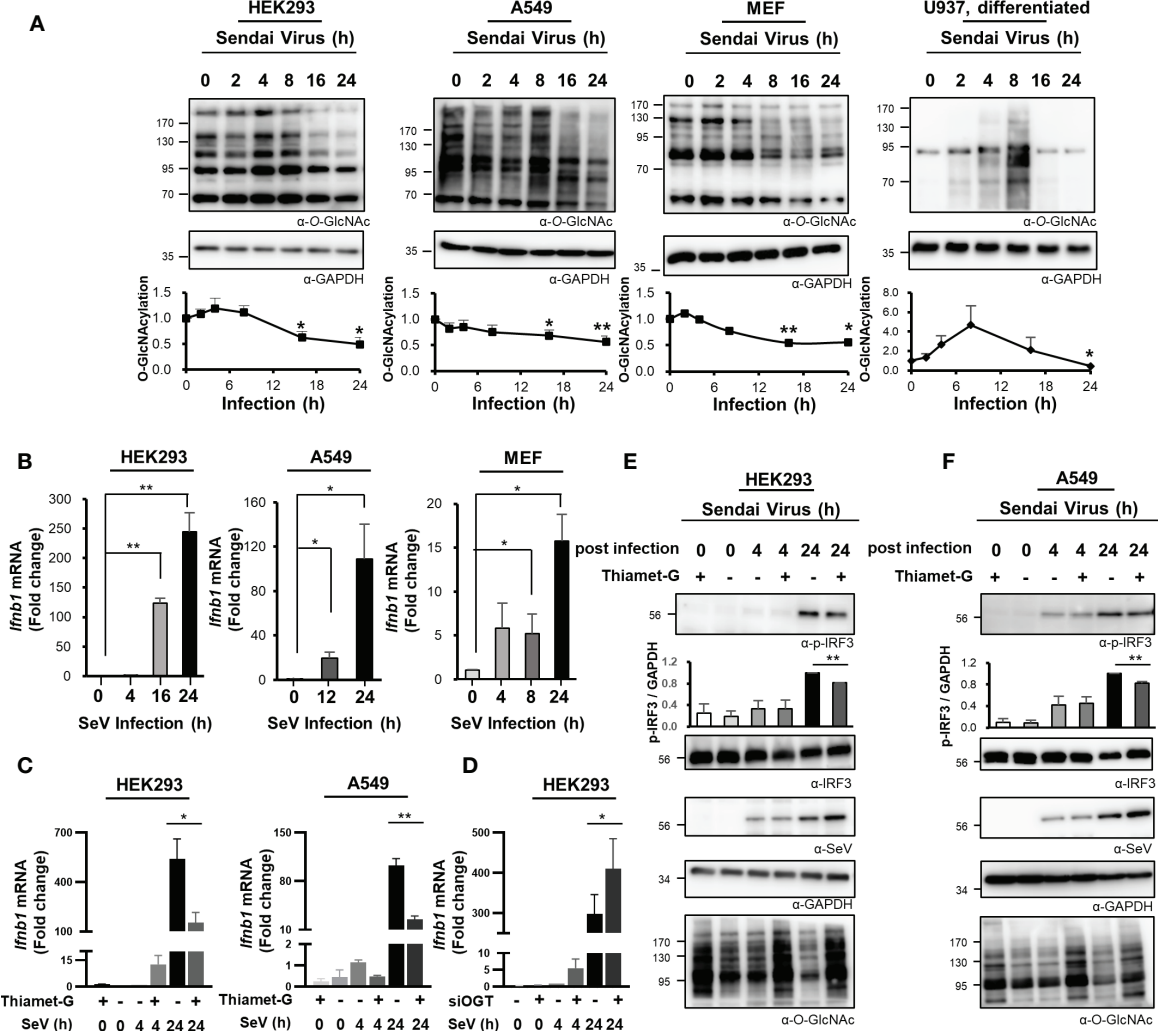
Reduced *O*-GlcNAcylation in response to RNA virus infection activates the innate immune response. **(A)** *O*-GlcNAcylation over time after infection with Sendai virus (SeV; 100 HAU at t = 0) in HEK293, A549, MEF, and PMA-differentiated U937 cells as detected via Western blot analysis. In the graphs, cellular *O*-GlcNAcylation was normalized to GAPDH. **(B)** Expression of IFN-β mRNA over time as measured by real-time qRT-PCR after infection of HEK293 (left), A549 (middle) and MEF (right) cells with SeV (100 HAU). **(C)** HEK293 (left) and A549 (right) cells were treated with 1 μM of Thiamet-G(+) or PBS (–) for 4 h before infection with SeV (100 HAU). Cells were collected at 4 and 24 h post-infection. Expression of IFN-β was measured by real-time qPCR. Statistical significance was determined by two-tailed student t-test. **(D)** HEK293 cells were transiently transfected with siCTL or siOGT for 48 h before infection with SeV (100 HAU). Cells were collected at 4 and 24 h post-infection. Expression of IFN-β was measured by real-time qPCR. **(E, F)** Under the same conditions as in **(C)**, phosphorylation of IRF3 (Ser-396-p-IRF3) was evaluated via Western blot. Replication of SeV in HEK293 (left) and A549 (right) was also evaluated via Western blot. Statistical significance was determined by two-tailed student t-test. All experiments were repeated at least three times. Each figure was statistically analyzed with the indicated n number. **(A)** HEK293 (left, n = 4) A549 (middle to left, n = 3) MEF (middle to right, n = 3) U937(right, n = 4) **(B)** HEK293 (left, n = 3) A549 (middle, n = 4) MEF (right, n = 3) **(C)** HEK293(left, n = 5) A549(middle, n = 3) **(D)** HEK293(n = 4) **(E)** HEK293(n = 3) **(F)** A549(n = 3). Data are presented as mean ± standard error (SEM); *p < 0.05, **p < 0.01.

The glucose concentration in cells is closely associated with the production of UDP-GlcNAc via the HBP. Lactate, a glucose intermediate, was recently identified as a suppressor of MAVS-mediated RLR signaling (22). OGT, an enzyme that promotes *O*-GlcNAc cycling and that uses UDP-GlcNAc as a substrate, is expressed in most tissues, including epithelial cells (23). *O*-GlcNAcylation in cells functions as both a nutrient and stress sensor (12). In this study, we evaluated changes in *O*-GlcNAcylation in various types of cells over time in response to infection with SeV; this is a single-stranded RNA virus pathogen that presents dsRNA intermediates to pattern recognition receptors in the cytosol. Global levels of *O*-GlcNAcylation were drastically reduced at the later time points in response to SeV infection of HEK293 and A549 cells (**Figure 1A**). The primary cell, MEF (mouse embryonic fibroblast) cells, also showed a decrease in *O*-GlcNAcylation (**Figure 1A**). Furthermore, we also confirmed that SeV infection resulted in elevated levels of intracellular *O*-GlcNAcylation in monocyte-macrophage U937 cells in response to differentiation with phorbol 12-myristate 13-acetate; these findings are consistent with those reported previously in which infection of macrophages with an RNA virus resulted in a substantial increase in intracellular *O*-GlcNAcylation (**Figure 1A**) (9, 10). As shown in the present study, elevated levels of *O*-GlcNAcylation levels were detected only in U937 cells differentiated into macrophages (**Figure S1A**); by contrast, the global decline in *O*-GlcNAcylation levels was observed in both undifferentiated and differentiated U937 cells at the later time points after infection (**Figures 1A**, **S1A**). Furthermore, cells of the breast epithelial line MDA-MB-231 and the colon epithelial line HT-29 also responded to SeV infection with reduced levels of *O*-GlcNAcylation (**Figure S1A**). In order to confirm that reduced *O*-GlcNAcylation levels in the results presented above are due to host immune responses rather than direct effects of Sendai virus itself, we treated HEK293 and A549 with poly(I:C) and then observed the *O*-GlcNAcylation levels at later points; however there was no change (**Figure S1B**). Therefore, we hypothesized that a global decrease in cellular *O*-GlcNAcylation during the later phases of SeV infection is a phenomenon common to many cell targets. Remarkably, we also confirmed that IFN-β expression reached its peak in both HEK293 and A549 cells at 24 h of SeV infection (**Figure 1B**). In addition, MEF cells showed the highest IFN-β expression at 24 h, which is consistent with the expression in HEK293 and A549 (**Figure 1B**). IFN-β is a major type I interferon that is synthesized and secreted in response to infection that promotes viral clearance and host immunity. Therefore, we speculated that the decrease in cellular *O*-GlcNAcylation observed at 24 h of infection may have a direct impact on IFN-β expression. To examine this hypothesis, we measured IFN-β mRNA levels in SeV-infected HEK293 and A549 cells that were treated with the OGA inhibitor, Thiamet-G, using real-time reverse transcription–polymerase chain reaction (RT-PCR). The results revealed that transcription of IFN-β was inhibited in response to increased levels of cellular *O*-GlcNAcylation achieved by treatment with Thiamet-G (**Figure 1C**). We conversely inhibited OGT activity through siRNA to demonstrate the importance of OGT in the inhibition of IFN-β expression. As a result, it was confirmed that the expression level of IFN-β increased in HEK293 when OGT was knocked down (**Figure 1D**). Under the same conditions as in **Figure 1C**, Thiamet-G introduced 24 h after SeV infection of HEK293 and A549 cells resulted in reduced phosphorylation of IRF3 by ~19% and ~17%, respectively, in association with amplified replication of SeV (**Figures 1E, F**). These results suggest that reduced levels of *O*-GlcNAcylation observed during the later stages of infection with an RNA virus serves to promote the innate immune response.

### Host Cell-Mediated Downregulation of *O*-GlcNAc Transferase Transcription in Response to Sendai Virus Infection

**Figure 2:**
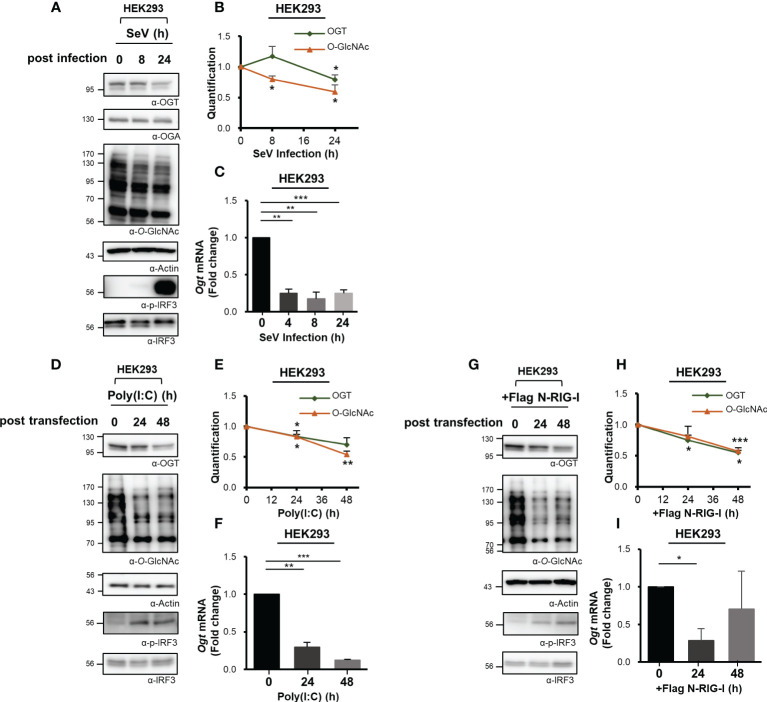
Downregulated transcription of *O*-GlcNAc Transferase (OGT) in response to virus infection. **(A)** Immunoreactive OGT was evaluated via Western blot analysis in HEK293 cells infected with SeV (100 HAU) at the time points indicated. **(B)** Expression of OGT and *O*-GlcNAc as shown in **(A)** was normalized to actin or GAPDH. **(C)** Real-time qPCR was performed to measure OGT mRNA expression levels. **(D)** 10 μM Poly (I:C) was transfected into HEK293 cells which were evaluated at the times indicated. Western blot analysis was performed to detect immunoreactive OGT protein. **(E)** OGT and *O*-GlcNAc as shown in **(D)** were normalized to levels of actin or GAPDH. **(F)** Real-time qPCR was performed to measure OGT mRNA expression levels. **(G)** To activate RLR signaling, the N-RIG-I plasmid was used to transfect HEK293 cells; cells were collected at the times indicated to measure OGT protein and mRNA expression levels. **(H)** Expression levels of OGT and *O*-GlcNAc in **(G)** were normalized to actin or GAPDH. All experiments were repeated at least three times. **(I)** Real-time qPCR was performed to measure OGT mRNA expression levels. Each figure was statistically analyzed with the indicated n number. **(A)**, **(B)** n = 4, **(D, E, G, H)** n = 3 and **(C, F, I)** n = 3. Data are presented as mean ± SEM; *p < 0.05, **p < 0.01, ***p < 0.001.

Cellular levels of *O*-GlcNAcylation decreased at 24 h after SeV infection (**Figure 1A**). Therefore, we examined the expression of immunoreactive OGT protein at various times after infection as indicated. These observations revealed that the levels of OGT protein decreased drastically in response to the strong induction of the innate immune response (**Figures 2A, B**, **S2A**). To investigate this observation further, OGT mRNA levels were measured by real-time RT-PCR. Surprisingly, we found that transcription of OGT decreased dramatically at all time points examined, most notably at the earliest time point after infection (i.e., 4 h) (**Figures 2C**, **S2B**). Given that some viruses alter the host transcriptome to facilitate their own replication and proliferation, these results raised additional questions as to whether the decrease in OGT transcription was driven directly by the virus. To address this question, we transfected cells with poly(I:C), which is a ligand capable of activating RLR-mediated signaling in the absence of an overt virus infection. Similar to SeV infection, transfection with poly(I:C) also promoted a decrease in OGT protein levels (**Figures 2D, E**, **S2C**). In addition, in MEF primary cells and HDF (Human dermal fibroblast) primary cells, OGT protein levels decreased in response to poly(I:C) transfection (**Figure S2D**). Furthermore, levels of OGT mRNA were also significantly reduced in cells transfected with poly(I:C) (**Figures 2F**, **S2E**). As such, we inferred that the host response, rather than the virus per se, promoted the observed reductions in OGT gene transcription. This conclusion was supported by findings associated with the overexpression of activated (N-)RIG-I. Overexpression of N-RIG-I has been reported to promote MAVS activation (24); N-RIG-I DNA constructs were transfected into HEK293 cells, resulting in a decrease in OGT protein and mRNA levels; these findings are consistent with the results shown in **Figures 2D, F** (**Figures 2G, I**).

### *O*-GlcNAcylation Directly Regulates Mitochondrial Antiviral Signaling Protein-Mediated Expression of Interferon-β

**Figure 3:**
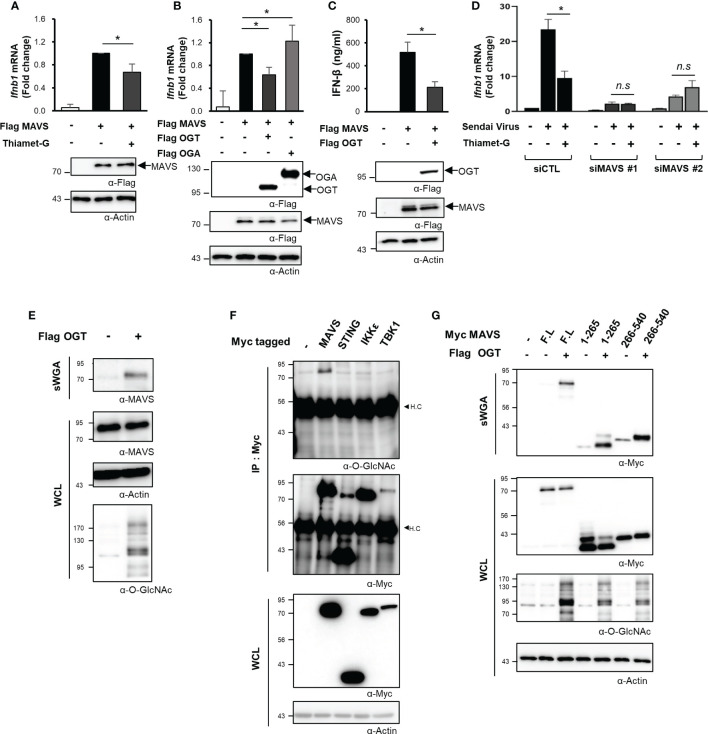
*O*-GlcNAcylation directly regulates MAVS-mediated expression of IFN-β. **(A)** IFN-β mRNA was measured by real-time qPCR was conducted to measure mRNA expression levels under conditions that mimicked viral infections in HEK293 cells by treating with 1 μM Thiamet-G for 24 h before induction of MAVS overexpression via transfection of a Flag-MAVS expression plasmid. **(B)** IFN-β mRNA expression induced by MAVS overexpression under conditions mimicking virus infection was measured by real-time qPCR together with co-overexpression of OGT and OGA. **(C)** An ELISA assay was conducted to compare secretion of IFN-β in response to OGT overexpression with that observed in response to MAVS overexpression (control condition). **(D)** Knock-down of MAVS in HEK293 cells was performed via transfection with MAVS-targeting siRNAs and examined after 48 h. Inhibition of SeV-induced IFN-β production in response to treatment with Thiamet-G was not observed in cells with MAVS knock-down. *n.s* indicated not statistically significant. **(E)** Precipitation using the lectin, sWGA, was conducted to demonstrate *O*-GlcNAcylation of endogenous MAVS and to examine the increase in MAVS *O*-GlcNAcylation in response to OGT overexpression. 1.5—2 mg of whole cell lysates (WCLs) obtained from HEK293 were incubated with agarose-conjugated sWGA overnight at 4^o^C. Purified proteins in sWGA precipitates were eluted with 2× SDS loading buffer then analyzed via Western blotting. **(F)** IP and Western blots were performed to identify *O*-GlcNAcylated proteins associated with RLR signaling. 1.5—2 mg of WCLs obtained from HEK293 were incubated with agarose-conjugated anti-Myc antibodies for 2 h at 4°C. Purified proteins in IP precipitates were eluted with 2× SDS loading buffer and then analyzed via Western blotting. Of the major molecules involved in RLR signaling, only MAVS was subject to *O*-GlcNAc modification. H.C means heavy chain. **(G)** Precipitation with sWGA was to confirm the *O*-GlcNAcylation of the N- or C-termini of MAVS. Plasmids promoting overexpression of MAVS 1-265 and 266-540 were transfected and evaluated after 24 h with or without OGT. 1.5 to 2 mg of WCLs obtained from HEK293 were incubated with agarose-conjugated sWGA overnight at 4°C. Purified proteins in SWGA precipitates were eluted with 2× SDS loading buffer then analyzed via Western blotting. The 1–265 and 266–540 regions are both modified with *O*-GlcNAc. All experiments were repeated at least three times. **(A–G)** – indicated cells transfected with flag or myc empty vectors. Each figure was statistically analyzed with the indicated n number. **(A)** n = 6, **(B)** n = 5, **(C)** n = 3, **(D)** n=3. Statistical significance was determined by two tailed student t-test. Data are presented as mean ± SEM; *p < 0.05, **p < 0.01.

Given the decline in *O*-GlcNAcylation in various cell types and the resulting impact on RLR-mediated signaling and the downstream production of IFN-β, we then aimed to identify the specific mechanisms underlying this response. MAVS is a powerful immune sensor that promotes innate immune responses and stimulates the production of IFN-β (25, 26). We overexpressed MAVS in HEK293 cells to induce a condition that mimicked viral infection (5). Interestingly, the IFN-β mRNA expression induced in response to MAVS overexpression dropped significantly in cells treated with Thiamet-G (**Figure 3A**). Furthermore, the expression of IFN-β mRNA was inhibited when MAVS and OGT were both overexpressed. By contrast, expression of IFN-β mRNA induced by overexpression of MAVS overexpression increased even further when cellular *O*-GlcNAcylation was reduced by co-overexpression of OGA (**Figure 3B**). The same findings were observed in assays designed to detect IFN-β protein synthesis and secretion (**Figure 3C**). On the basis of these results, we hypothesized that cellular *O*-GlcNAcylation may have a direct role in the regulation of MAVS activity, notably upon activation of RLR signaling. To examine this hypothesis, MAVS knock-down was achieved using targeted siRNAs (**Figure S3A**). As shown in **Figure 3D**, Thiamet-G treatment inhibited the expression of IFN-β in response to SeV infection in the control group but in cells transfected with siMAVS. As such, we concluded that *O*-GlcNAcylation regulates RLR signaling in a MAVS-dependent manner.

### Mitochondrial Antiviral Signaling Protein Is a Direct Target of *O*-GlcNAc Transferase

O-GlcNAc transferase (OGT) catalyzes the *O*-GlcNAcylation of numerous mitochondrial proteins (12). As such, we evaluated *O-*GlcNacylation of endogenously expressed MAVS via precipitation studies conducted with succinylated-wheat germ agglutinin (sWGA). Using this approach, we found that endogenous MAVS was *O*-GlcNAcylated (**Figure 3E**). Furthermore, recombinant (overexpressed) MAVS was also *O*-GlcNAcylated; *O*-GlcNAcylation of this target was increased in response to OGT overexpression (**Figures 3E**, **S3B**). We also evaluated *O*-GlcNAcylation of other intracellular proteins including STING, which is another immune sensor, as well as IKKϵ, a TBK1-associated factor involved in the innate immune response. Despite these efforts, our results indicated that MAVS alone was targeted for *O*-GlcNAcylation (**Figure 3F**). Even when IKKϵ or TBK1 was overexpressed with Flag tagged MAVS to activate MAVS signaling, we could not detect *O*-GlcNAcylation of IKKϵ or TBK1 (**Figure S3C**). We divided the 540-amino acid sequence of MAVS into N-terminal (1–265) and C-terminal (266–540) fragments to identify specific sites of *O*-GlcNAcylation. Full-length MAVS, the MAVS N-terminal fragment, and the MAVS C-terminal fragment were all overexpressed in HEK293 cells; *O*-GlcNAcylation of each polypeptide was investigated by sWGA-mediated precipitation. Significantly, we detected *O*-GlcNAcylation of both N- and C-terminal fragments of MAVS (**Figure 3G**). These results clearly indicate that there are potential *O*-GlcNAcylation sites in the N-terminal region of MAVS besides that previously identified (i.e., Serine 366) in its C-terminal region (9).

### Mitochondrial Antiviral Signaling Protein Contains a Heavily *O*-GlcNAcylated Serine-Rich Region That Can Inhibit RIG-I-Like Receptors-Mediated Signaling

**Figure 4:**
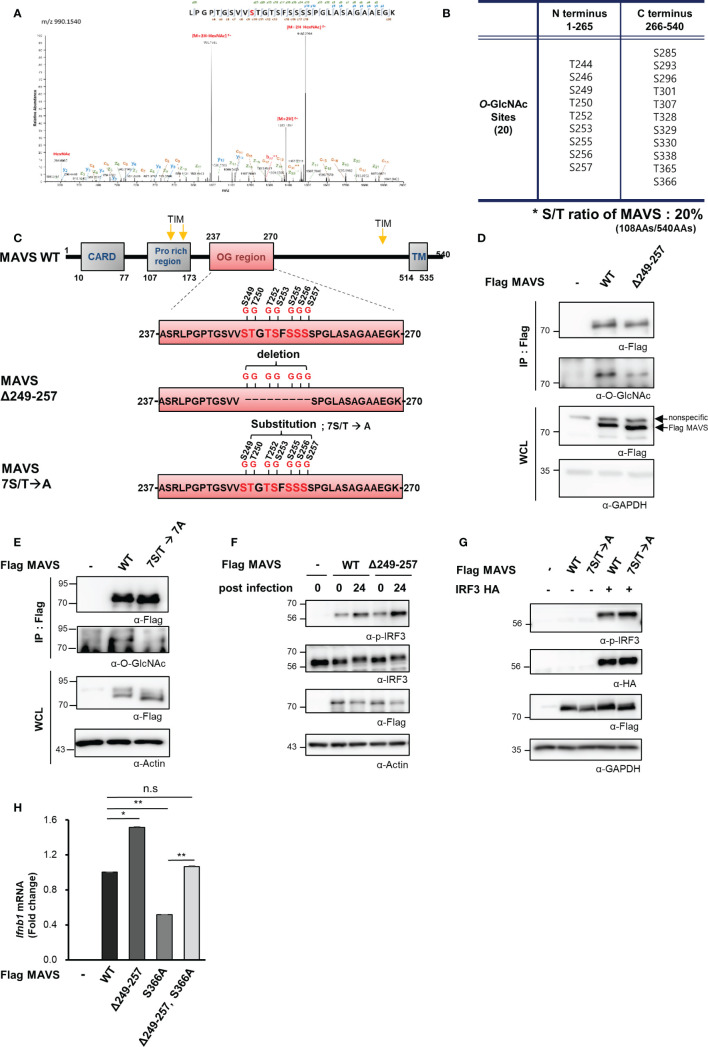
MAVS contains a heavily O-GlcNAcylated serine-rich region that inhibits RLR signaling. **(A)** Fusion M/S analysis was conducted to identify sites of O-GlcNAcylation in MAVS. EThcD spectra of the O-glylcopeptide LPGPTGSVVSTGTSFSSSSPGLASAGAAEGK from human MAVS is as shown. The site of O-GlcNAc modification was identified as serine 249. The y, b, C, and z fragments detected are as indicated in the sequence. **(B, C)** MAVS O-GlcNAcylation sites and the protein structures of the wild-type and MAVS Δ249–257 mutant. **(D, E)** IP and Western blots were performed to compare the O-GlcNAcylation of wild-type MAVS with **(D)** the mutant in which the serine-rich region including the O-GlcNAcylation sites were detected and **(E)** the mutant with the 7S/T to alanine substitution. The pRK5-flag-tagged MAVS wild-type and mutant plasmids were transfected into HEK293 cells and evaluated 24 h later. WCLs were incubated with agarose-conjugated anti-FLAG antibody (M2) for 1–2 h at 4°C. **(F, G)** Western blot analysis of phospho-IRF3 (p-IRF3) in WCLs from HEK293 cells. The p-IRF3 levels were determined by transfection with pRK5-flag-tagged MAVS wild-type, **(F)** deletion mutant, or **(G)** substitution mutant plasmids followed by evaluation 24 h later; cells were infected with SeV (100 HAU) for 24 h followed by Western blotting. **(H)** Expression of IFN-β mRNA induced by overexpression of wild-type and mutant MAVS was measured by real-time qPCR. The decreased levels of IFN-β expression in response to the S366A mutant underwent full recovery in response to the Δ249–257 mutant containing S366A when compared to the wild type. n = 3. **(D–H)** Experiments were performed at least three times. **(D–H)** – indicated cells transfected with flag or myc empty vectors. Statistical significance of **(H)** was determined by one-way ANOVA. Results were presented as mean ± SEM; *p < 0.05, **p < 0.01.

*O*-GlcNAcylation takes place at hydroxyl groups found on serine and threonine residues. Notably, serine and threonine represent 20% of the amino acids (108 of the total 540) in the MAVS polypeptide. *O*-GlcNAcylation sites within MAVS were identified by fusion mass spectrometry (Fusion M/S). A total of 20 *O*-GlcNAcylation sites were identified by this method (**Figures 4A–C**, **S4AF**). Remarkably, seven *O*-GlcNAcylation sites that were adjacent to one another were identified in a serine-rich region within the aforementioned N-terminal fragment of MAVS (**Figure 4C**). To evaluate the function of these *O*-GlcNAc modifications, we generated a MAVS mutant that deleted nine amino acids, including the seven potential *O*-GlcNAcylation sites (Δ249–257); *O*-GlcNAcylation levels were explored in HEK293 cells that overexpressed both the wild-type and the MAVS Δ249–257 deletion mutant. Consistent with the fusion M/S results, the *O*-GlcNAcylation levels of the MAVS Δ249–257 mutant were significantly lower than those detected in the MAVS wild-type polypeptide (**Figure 4D**). *O*-GlcNAcylation levels were also dramatically reduced in the 7S/T→7A substitution mutant in which all seven *O*-GlcNAcylation sites were substituted with alanine (**Figure 4E**). As such, we hypothesized that *O*-GlcNAcylation at the serine-rich region of MAVS would have an impact on IFN-β production in response to SeV infection. First, we examined phosphorylation of IRF3 to determine whether reduced *O*-GlcNAcylation in the MAVS Δ249–257 mutant had an impact on RLR-mediated signaling. We found that phosphorylation of IRF3 was increased in HEK293 cells transfected with MAVS Δ249–257 over that observed in response to MAVS wild-type when cells were infected with SeV (**Figure 4F**). Consistent with the results observed in HEK293 cells transfected with the MAVS Δ249–257 mutant, phosphorylation of IRF3 was further enhanced in cells transfected with the 7S/T→7A substitution mutant in response to SeV infection (**Figure 4G**). Furthermore, expression of IFN-β mRNA was also significantly elevated in cells transfected with the MAVS Δ249–257 mutant compared to the wild type (**Figure 4H**). Finally, and consistent with previous reports that describe *O*-GlcNAcylation at MAVS Serine 366 and its role in promoting RLR-mediated signaling, decreased expression of IFN-β was observed in cells transfected with a MAVS S366A mutant. Surprisingly, when compared to the results obtained from cells transfected with the MAVS S366A mutant, we observed full recovery of IFN-β expression upon deletion of the serine-rich 249–257 region from the S366A mutant. Collectively, these results suggest that *O*-GlcNAcylation at the serine-rich region of MAVS (amino acids 249–257) modulates MAVS-activated RLR-mediated signaling via a mechanism that is distinct and different from that associated with *O*-GlcNAcylation of S366.

### Mitochondrial Antiviral Signaling Protein *O-*GlcNAcylation Interferes With the Formation of Its Aggregates

**Figure 5:**
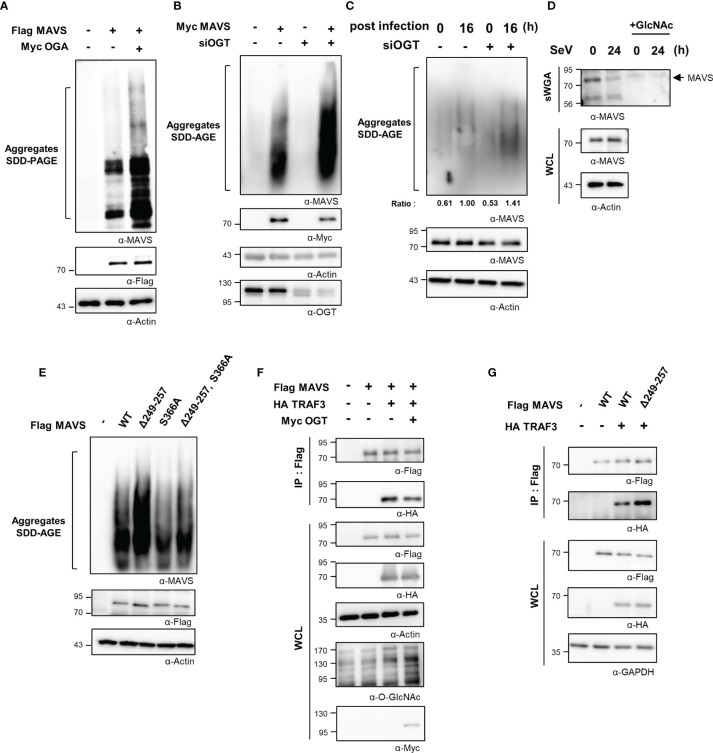
O-GlcNAcylation of MAVS interferes with aggregate formation and interactions with TRAF3. **(A)** Non-reducing SDD-PAGE analysis was conducted to determine the level of MAVS aggregation. MAVS overexpression in HEK293 cells induced aggregate formation levels with or without overexpression of OGA. **(B)** Knock-down of OGT in HEK293 cells was detected at 48 h after transfection with specific siRNAs; this was performed before induction of MAVS overexpression for 24 h, after which SDD-AGE analysis was performed. **(C)** Endogenous MAVS aggregates were detected by SDD-AGE under conditions of OGT knock-down. HEK293 cells were transfected with siOGT; 48 h later, cells were infected with SeV (100 HAU) and evaluated at 16 h after infection. MAVS aggregates were normalized to the expression level of endogenous MAVS. **(D)** Precipitation was performed with sWGA followed by Western blot analysis to detect the O-GlcNAcylation levels of MAVS after a longer period of time after SeV infection (100 HAU). Cells were infected with SeV for 24 h before collection. WCLs were incubated with sWGA-conjugated beads overnight at 4°C and then were eluted for Western blot analysis. **(E)** An SDD-AGE assay was conducted to compare the levels of aggregation of wild-type MAVS and mutants with deleted O-GlcNAcylation sites. The pRK5-flag-tagged MAVS wild-type and mutant (Δ249–257, S366A, Δ249–257 containing S366A) plasmids were used to transfect HEK293 cells and were evaluated after 24 to 48 h. **(F)** A co-IP assay was performed to evaluate the interactions between MAVS and TRAF3. The pRK5-flag-tagged MAVS and HA-TRAF3 expression plasmids co-overexpressed in HEK293 cells both with and without Myc-OGT overexpression. **(G)** A co-IP assay was performed to evaluate the interactions between wild-type or the Δ249–257 mutant MAVS and HA-TRAF3 in HEK293 cells. Expression plasmids were used to transfect cells that were evaluated at 24 to 48 h. All experiments were repeated at least three times. **(A–G)** – means cells transfected with flag or Myc or HA empty vectors.

Thus far, we have demonstrated *O*-GlcNAcylation of the MAVS 249–257 serine-rich region results in down-regulated RLR signaling infection with SeV. We further examined the mechanism by which *O*-GlcNAcylation at this site suppresses the IFN-β response. Full activation of MAVS requires self-aggregation at the mitochondrial outer membrane. The aggregation and resolution responses of MAVS are strongly regulated by post-translational modifications, including ubiquitination (21, 27, 28). Results from earlier studies indicated that *O*-GlcNAcylation inhibited the formation of prion-like aggregates of proteins that include α-synuclein and tau (29, 30); as such, its role in preventing the formation of critical MAVS aggregates was explored. Intriguingly, upon co-overexpression of both MAVS and OGA, MAVS could form aggregates as revealed in a semi-denaturing detergent-polyacrylamide gel electrophoresis (SDD-PAGE) assay (**Figure 5A**). Furthermore, the degree of MAVS aggregation increased drastically in response to OGT knock-down (**Figures 5B**, **S5A**). Consistent with these results, endogenously generated MAVS aggregates were detected at higher levels in cells subjected to OGT knock-down during the later stages of SeV infection (at 16 h; **Figure 5C**). To exclude the possibility that the observed increase in MAVS aggregation was due to changes in MAVS protein stability that might result from reduced levels of *O*-GlcNAcylation, MAVS protein levels were evaluated in HEK293 and A549 cells treated with cycloheximide. Under these conditions, MAVS stability was not diminished in the presence or absence of Thiamet-G (**Figures S5B**, **S5C**). *O*-GlcNAcylation of endogenously expressed MAVS was found to have decreased at 24 h after SeV infection (**Figures 5D**, **S5D**). GlcNAc competition studies revealed that *O*-GlcNAcylation MAVS was significantly reduced under these conditions (**Figure 5D**). We then examined the degree of MAVS aggregation in cells overexpressing the MAVS Δ249–257 deletion mutant. As expected, the degree of aggregation was higher in cells overexpressing the MAVS Δ249–257 deletion mutant than that observed in cells overexpressing the MAVS wild type (**Figure 5E**); interestingly, we observed no increase in the degree of aggregation when comparing responses of the MAVS wild-type protein to that of the S366A mutant. Nevertheless, we were still able to observe the increased degree of aggregation in the deletion of the serine-rich 249–257 from the S366A mutant compared to the S366A mutant (**Figure 5E**). These results suggest that *O*-GlcNAcylation of MAVS specifically at the serine-rich region (249–257), restricts the formation of MAVS aggregates.

### Mitochondrial Antiviral Signaling Protein *O-*GlcNAcylation Interferes With Its Interaction With TRAF3

The sequence of MAVS contains several TRAF-interacting motifs (TIMs) that facilitate its interactions with these signaling molecules. The interaction between MAVS and TRAFs is critical to activate MAVS-mediated downstream signaling. TRAF3 is recruited to aggregates of MAVS and thereby promotes the activation of IRF3 via the K63-ubiquitination of MAVS. The interaction between MAVS and TRAF3 was examined through co-immunoprecipitation (co-IP) studies designed to identify the mechanism by which *O*-GlcNAcylation of MAVS inhibits expression of IFN-β. The interaction between MAVS and TRAF3 was inhibited in cells overexpressing OGT (**Figure 5F**). Furthermore, we found that the interaction between TRAF3 and MAVS Δ249–257 was significantly enhanced compared with its interaction with wild-type MAVS (**Figure 5G**). Since there are previous reports that *O*-GlcNAcylation of MAVS regulates ubiquitination of MAVS, we needed to confirm whether *O*-GlcNAcylation at the serine-rich region (amino acids 249–257) also regulates ubiquitination of MAVS (9, 10). We confirmed that MAVS ubiquitination was slightly increased in MAVS Δ249–257 deletion mutant compared to wild type (**Figure S5E**). As such, we concluded that *O*-GlcNAcylation at the serine-rich region of MAVS specifically inhibits RLR signaling by interfering with the interaction between MAVS and TRAF3. Taken together, these findings suggest that *O*-GlcNAcylation is involved in modulating host defense mechanisms.

## Discussion

**Figure 6:**
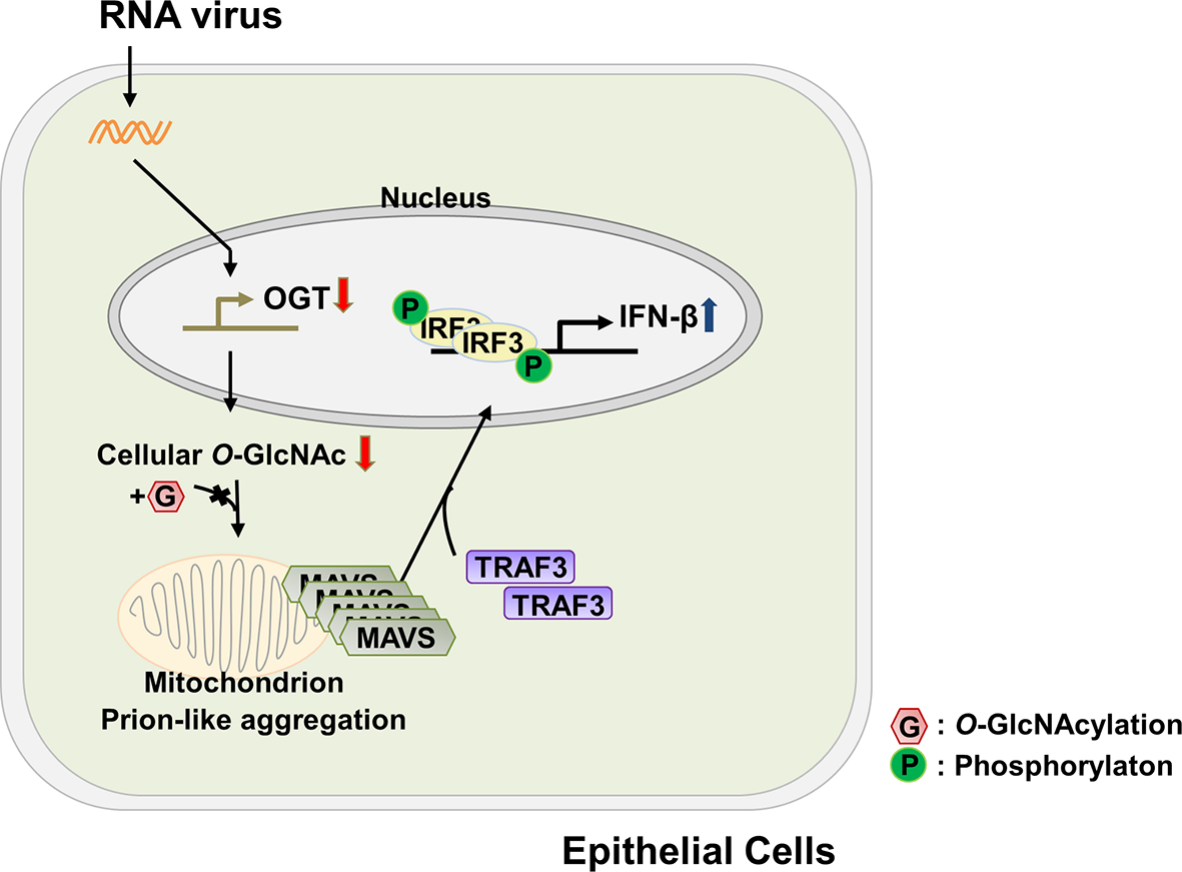
Schematic of the proposed model depicting *O*-GlcNAcylation of MAVS at S249-S257 and its role in RLR-mediated signaling. The functional mechanism underlying cellular *O*-GlcNAcylation of MAVS and its role in promoting RLR-mediated signaling is described in **Figure 6**.

Dysregulated energy metabolism is directly linked to the pathogenesis of numerous diseases; an imbalance in energy metabolism has also been found to disrupt the host defense system (31, 32). In recent years, attempts have been made to identify a role for glucose metabolism, the central pathway associated with energy metabolism, in modulating one or more aspects of the innate immune response. As but one example, several groups have examined the relationship between glucose metabolism and RLR-mediated signaling. Among these findings, RLR-induced production of type-I IFNs was observed under conditions in which glucose metabolism was inhibited and that cells maintained in a low-glucose environment could produce higher levels of IFN than cells maintained in a high-glucose medium (22). By contrast, results from another study revealed that activation of RLR-mediated signaling resulted in an increase in glucose metabolism (9). These conflicting results will most certainly be important toward the effort to understand the full nature of the cross-talk between the innate immune response and glucose metabolism. In this study, we have demonstrated that levels of *O*-GlcNAcylation, which are known to respond rapidly to extracellular stimuli (33), decrease significantly in response to infection with an RNA virus; this ultimately leads to innate immune responses in target epithelial cells at later time points during infection that are associated with production of IFN-β. The functional mechanisms underlying cellular *O*-GlcNAcylation and their role in promoting RLR-mediated signaling are as shown in **Figure 6**. The host cell responds to virus infection by shutting down the expression of OGT. This response results in decreased levels of OGT and thus global depletion of cellular *O*-GlcNAcylation. This response has a specific impact on *O*-GlcNAcylation at the serine-rich region (amino acids 249–257) of MAVS; this promotes MAVS aggregation and enhances the interactions between MAVS and TRAF3. Because of these interactions, IRF3 is phosphorylated, undergoes dimerization, and is translocated into the nucleus where it promotes IFN-β transcription and thus activates IFN-β-mediated host defense responses. Taken together, the results presented in this study reveal that *O*-GlcNAcylation is a key link between glucose metabolism and the innate immune response.

Our study elucidated an important role for *O*-GlcNAcylation of MAVS; this post-translational modification resulted in suppression of RLR-mediated signaling in epithelial cells via a mechanism that was clearly different and distinct from those previously reported for MAVS Ser366 or for the seven associated sites at amino acids 322–347 within the MAVS polypeptide (9, 10). In these previous studies, O-GlcNAcylation of MAVS resulted in increased IFN-β production at relatively early time points after viral, similar to those identified for the IFN-β response of macrophages. However, in the case of epithelial cells, the time IFN-β production is typically observed at later time points when compared with those observed in infected macrophages (**Figure 1B**). We identified an unexpected decreased in the transcription of OGT in response to virus infection; this resulted in the global reduction of cellular O-GlcNAcylation and the specific reduction of the O-GlcNAcylation of MAVS. Our study revealed a mode of action that explains how *O*-GlcNAc modification modulates protein function in a site-specific manner. Furthermore, this mechanism suggests that *O*-GlcNAcylation, similar to phosphorylation, may serve as a central communicator promoting immune-mediated signal transduction.

The results of our study revealed drastic reductions in mRNA encoding OGT at early time points following SeV infection (**Figure 2**). This decline in OGT mRNA was not a response to specific viral proteins but appears to be modulated by host defense mechanisms. As such, we determined that the host response itself regulates transcription of OGT. Therefore, we proceeded to determine how the observed reduction of OGT mRNA was ultimately linked to the activation of RLR-mediated signaling. Given the recent reports indicating that OGT mRNA stability could be modulated by specific miRNAs (34, 35), we inferred that alterations of OGT might relate to specific post-transcriptional alterations that could promote mRNA decay. Further investigation will be needed to determine a role for virus-mediated activation of negative TFs and transcriptional co-repressors of OGT.

Some viruses are capable of sequential expression of immediate early (IE), early, and late viral genes that serve to support of their replication and propagation (36). *O*-GlcNAcylation of host transcriptional factors that promote viral gene expression, including HCF-1 and Sp1, have also been reported (37–39). However, we are not aware of any reports that describe host-mediated regulation of OGT transcription in response to viral infection. As such, to the best of our knowledge, this is the first demonstration of transcriptional modulation of OGT mediated by virus-infected host cells that resulted in the reduction of *O*-GlcNAcylation and concomitant induction of an innate immune response. Moreover, it is important to discuss the role of *O*-GlcNAcylation of host TFs. For example, HCF1 is a host TF required for the transcriptional transactivation of the IE genes of HSV. *O*-GlcNAcylation of HCF1 promotes increased expression of HSV genes. When OGT activity is chemically inhibited by the OGT inhibitor, OSMI-1, late capsid formation of HSV and replication of both HSV and cytomegalovirus are inhibited (18). Furthermore, several of the TFs involved in the regulation of human immunodeficiency virus (HIV)-1 genes are modified by *O*-GlcNAcylation, including AP-1, yin-yang1 (YY1), NFATc1, NF-κB, and Sp1 (40–42). Among these findings, *O*-GlcNAcylation of Sp1 suppresses the long terminal repeat (LTR) region of HIV-1 and thereby inhibits HIV-1 replication (39). Besides the viral life cycle regulation via *O*-GlcNAcylation of host TFs, we have demonstrated that the virus infection results in alterations to the transcriptional patterns of the gene encoding the host OGT; we have shown that this is a host antiviral defense mechanism that ultimately leads to the continuous production of IFN-β.

In this study, we examined the functional significance of *O*-GlcNAcylation of the MAVS serine-rich region (249–257). However, the MAVS Δ249–257 deletion mutant still showed weak *O*-GlcNAcylation by OGT; this result suggested that there are likely to be additional *O*-GlcNAcylation sites on MAVS. Twenty potential MAVS *O*-GlcNAcylation sites were identified by Fusion M/S, including *O*-GlcNAcylation at Ser366 and a region including amino acids 324–347 on MAVS; these findings are consistent with those recently reported by Li et al (2018). and Song et al (2019).. The function of *O*-GlcNAcylation of Ser366 and the amino acids 324–347 region may be to promote RLR-mediate signaling in macrophages. However, we found that the aggregation and activity of MAVS were increased in epithelial cells with diminished levels of OGT and *O*-GlcNAcylation. Therefore, a more systematic approach will be needed to examine the nature of these differences and to identify their unique and important roles in promoting immune cell activation.

Additionally, our findings also confirmed that the viability of HEK293 and A549 cells was not significantly decreased within the 24 h after virus infection in as determined by MTT assays (**Figure S5F**). Ultimately, the absence of virus clearance mechanisms led to the death of host cells in response to the long-term infection. *O*-GlcNAcylation has protective effects concerning cell death; specifically, mechanisms that promote cell death also reduces the cellular levels of *O*-GlcNAcylation. However, cells that have been infected for a long period of time cannot activate IRF3 phosphorylation or produce IFN-β despite reductions in *O*-GlcNAcylation. As such, our study did present any reductions in *O*-GlcNAcylation associated with cell death and/or its impact on innate immune response.

MAVS activity is clearly regulated by the degree of *O*-GlcNAcylation as well as by processes including ubiquitination and phosphorylation (9, 21, 43–45). Furthermore, there is a yin–yang relationship between *O*-GlcNAcylation and phosphorylation. Interestingly, we also identified a phosphorylation site in MAVS (Serine 258). This phosphorylation site is very close to one of the *O*-GlcNAcylation sites identified in this study; as such, future studies will determine whether there are cooperative or competitive interactions between phosphorylation and *O*-GlcNAcylation at these sites. The results of this type of investigation will provide insight into how *O*-GlcNAcylation communicates with phosphorylation to modulate host defense mechanisms.

In summary, we demonstrated that MAVS-mediated RLR signaling, which is necessary for antiviral immune responses of epithelial cells, is inhibited by *O*-GlcNAcylation of MAVS, which restricts the formation of self-aggregates. *O*-GlcNAcylation of MAVS is also impaired via reduction of OGT mRNA expression, which is a newly discovered host defense mechanism that serve to accelerates innate immune responses. An improved understanding of *O*-GlcNAcylation and its role in RLR-mediated signaling in RNA virus-infected cells may present OGT as a potential therapeutic target for the treatment of patients with immune dysfunction.

## Data Availability Statement

The original contributions presented in the study are included in the article/**Supplementary Material**; further inquiries can be directed to the corresponding author/s.

## Author Contributions

JS and JC designed the study. JS and JC wrote the manuscript. JS performed most of the experiments. YP, TK, JK, and SS performed the experiments. YP assisted in editing the manuscript. HK, YS, MK, and EC identified the *O*-GlcNAcylation site of MAVS by using Fusion M/S. JC, WY, BP, JK, and YL supervised the study. All authors contributed to the article and approved the submitted version.

## Funding

This research was supported by the National Research Foundation of Korea (NRF). Grants were given to JC from the Korean Government (NRF-2016R1A5A1010764 and NRF-2015M3A9B6073840).

## Conflict of Interest

The authors declare that the research was conducted in the absence of any commercial or financial relationships that could be construed as a potential conflict of interest.
