# Supplementary material for: O-Linked N-Acetylglucosamine Modification of Mitochondrial Antiviral Signaling Protein Regulates Antiviral Signaling by Modulating Its Activity
Source: Front Immunol. 2021 Feb 2;11:589259. doi: 10.3389/fimmu.2020.589259 (PMC7884448; doi:10.3389/fimmu.2020.589259)
Supplement: Supplementary file 1 [file Table_1.docx]

Supplementary Material

## Supplementary Figures

**
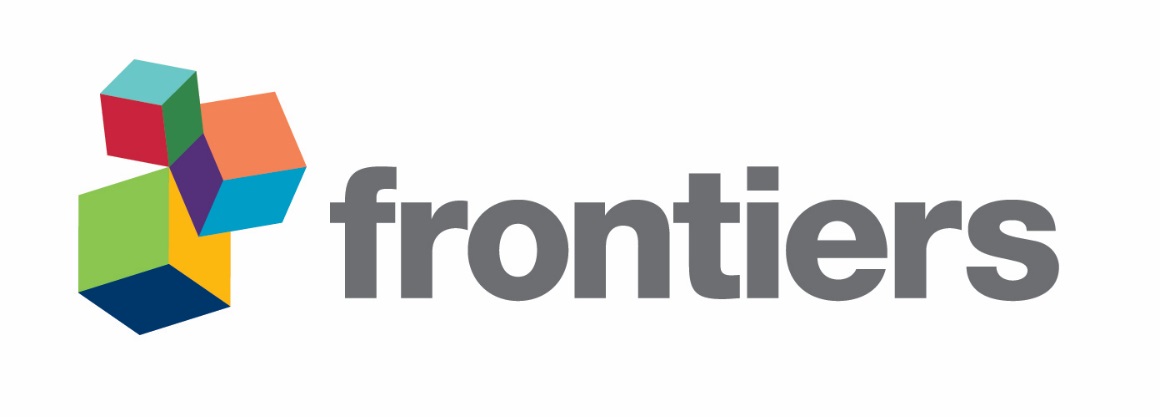
**


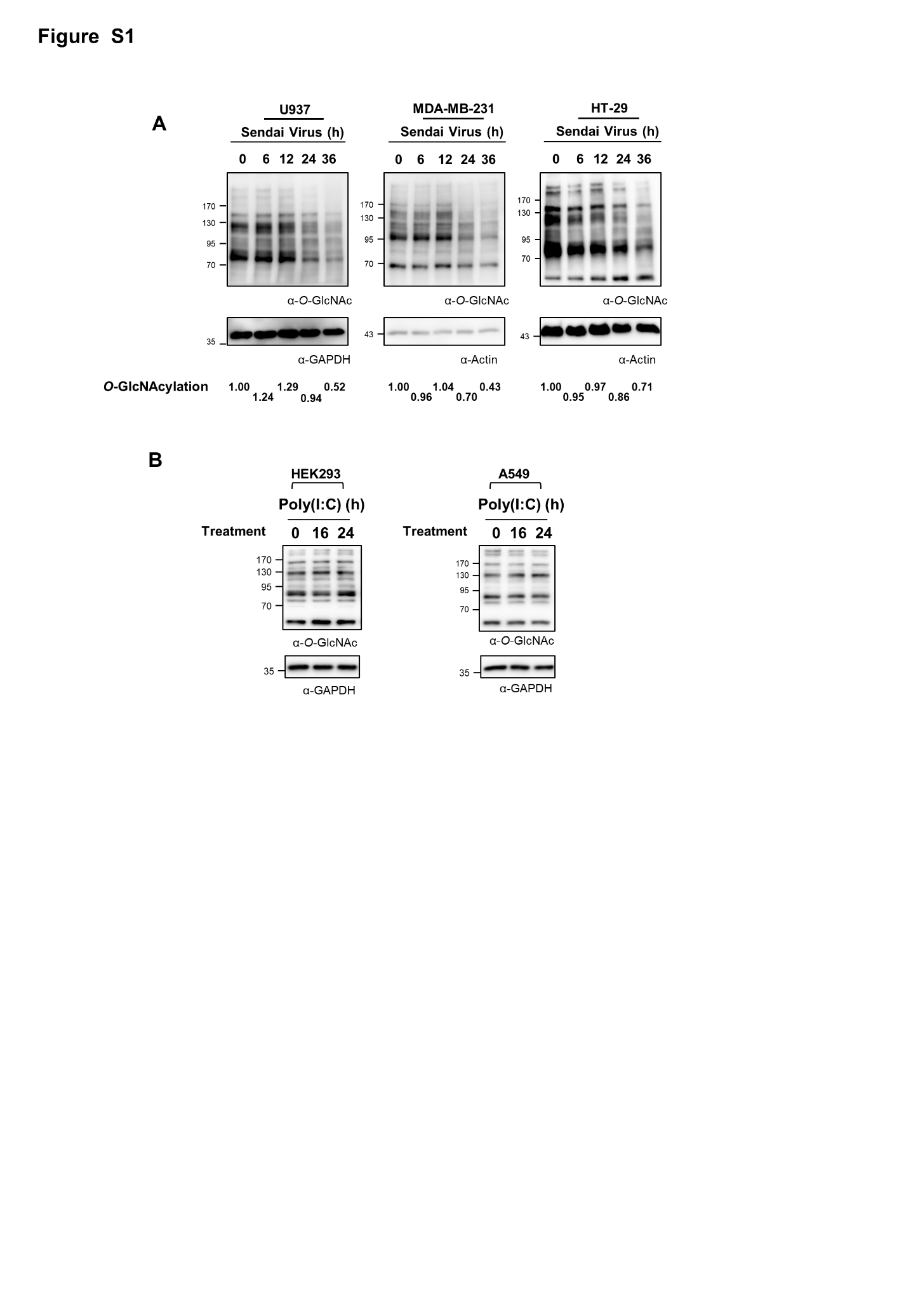


**Supplementary Figure 1.** **Reduced *O*-GlcNAcylation in various cell types detected response to RNA virus infection.**

(A) *O*-GlcNAcylation over time in response to SeV infection (100 HAU) in U937, MDA-MB-231, and HT-29 cells detected via Western blot. Cellular *O*-GlcNAcylation was normalized to GAPDH or to actin. (B) Poly(I:C) was treated to HEK293 and A549 cells at 0 hr; thereafter, *O*-GlcNAcylation levels were analyzed at later points via Western blot.

**
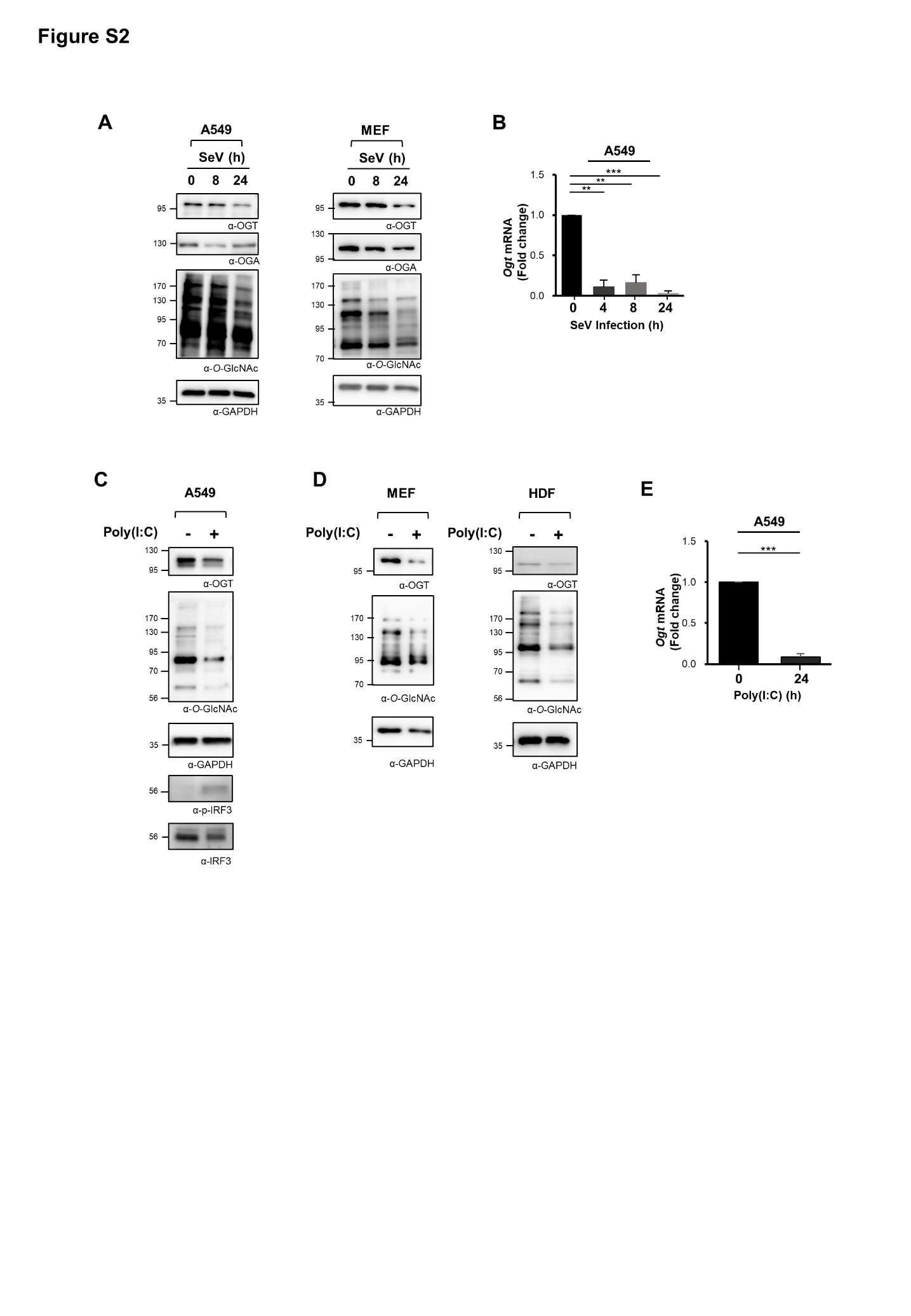
**

**Supplementary Figure 2.** **Transcription of OGT is down-regulated in host cells in response to activation of RLR-mediated signaling.**

(A) Western blot analysis was performed to detect immunoreactive OGT in A549, MEF cells infected with SeV (100 HAU) for 24 h. (B) Real-time qPCR was performed to evaluate expression of OGT mRNA in A549 cells. (C-D) 10 μM Poly (I:C) was transfected into A549 cells(C), MEF, and HDF cells(D) and evaluated at 24 h. Western blot analysis was performed to detect immunoreactive OGT. Western blot analysis was performed to detect immunoreactive OGT. (E) Real-time qPCR was performed to evaluate expression of OGT mRNA. Data are presented as mean ± SEM; **p*< 0.05, ***p*< 0.01, ****p*< 0.001.

**
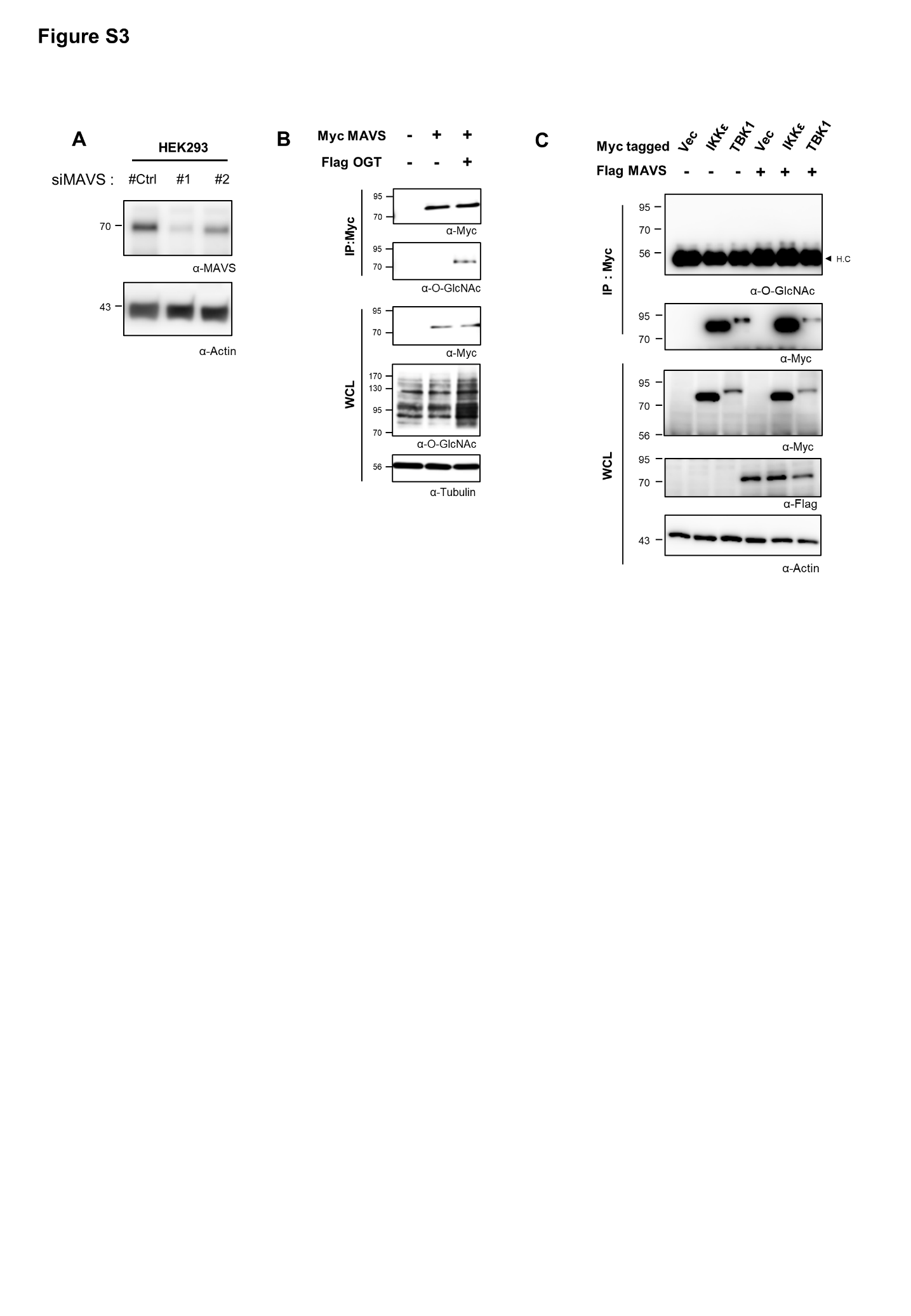
**

**Supplementary Figure 3. MAVS is *O*-GlcNAcylated by OGT.**

(A) Knock-down of MAVS was detected at 48 h after transfection with siMAVS in HEK293 cells using the Lipofectamine-RNAi max reagent. (B) IP and Western blot assay were performed to evaluate the extent of *O*-GlcNAcylation of MAVS overexpressed in HEK293 cells. (C) IP and Western blot assay were performed to confirm O-GlcNAcylation of IKKε or TBK1 overexpressed in HEK293 cells.

**
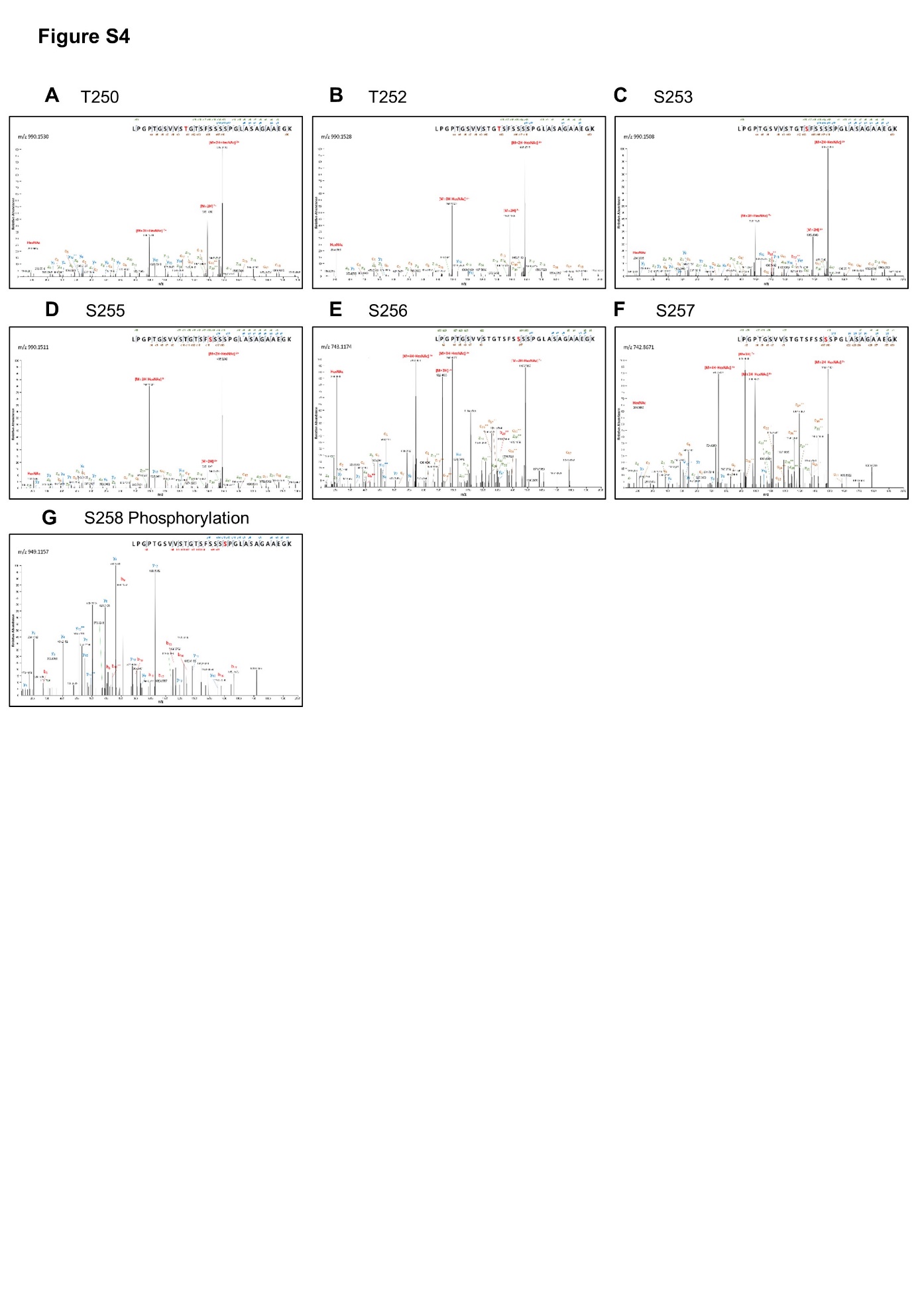
**

**Supplementary Figure 4. Fusion M/S analysis of MAVS *O*-GlcNAcylation sites.**

(A-F) Fusion M/S analysis was conducted to identify *O*-GlcNAcylation sites in MAVS. *O*-GlcNAcylation was detected in the MAVS serine-rich region (S249-S257). The EThcD spectrum of the O-glycosylated peptide LPGPTGSVVSTGTSFSSSSPGLASAGAAEGK from human MAVS is shown. The sites of *O*-GlcNAc modification are identified as (A) threonine 250, (B) threonine 252, (C) serine 253, (D) serine 255, (E) serine 256, and (F) serine 257. The y, b, C and z fragments detected are as indicated in the sequence. (G) Fusion M/S analysis was used to identify phosphorylation sites in MAVS adjacent to *O*-GlcNAcylation sites. The HCD spectrum of the phospho-peptide with respect to the parent mass of (m/z) 949.1157 defines a phosphorylation site at serine 258. The y and b fragments and the phosphorylation site (pS) are as indicated in the sequence of the peptide.

**
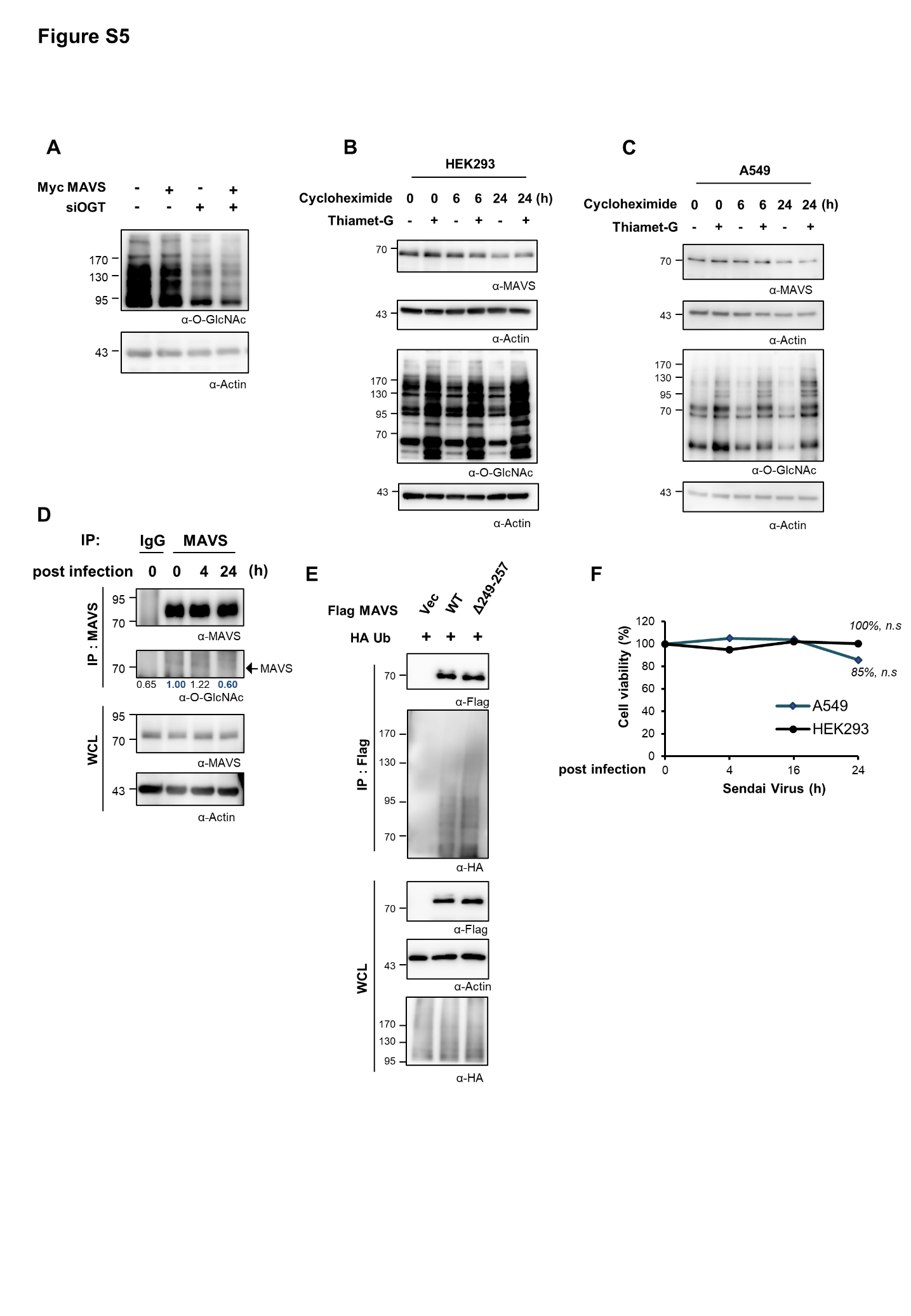
**

**Supplementary Figure 5. MAVS protein stability is not controlled by *O*-GlcNAcylation.**

(A) Knock-down of OGT was detected at 48 h after transfection of HEK293 cells with siOGT was using the Lipofectamine-RNAi max reagent. (B, C) Cycloheximide was introduced to evaluate changes in MAVS protein stability in response to treatment with Thiamet-G. Cells were plated 24 h before the addition of cycloheximide (final concentration of 10 μg/ml); Thiamet-G was added to a final concentration of 1 μM at 2 h before cycloheximide treatment. Cycloheximide treatment was conducted for the periods of time indicated in HEK293 (left) and A549 (right) cells. (D) IP and Western blot analysis were performed to detect *O*-GlcNAcylation of MAVS after SeV (100 HAU) infection. Cells were infected with SeV and harvested at 4 or 24 h. WCLs were incubated with anti-MAVS antibody for 24 h at 4°C followed by the addition of protein A/G-conjugated agarose and incubation for an additional 2 h at room temperature. Endogenous MAVS (faint band) was as indicated by arrows. To quantify O-GlcNAcylation level of MAVS, anti-O-GlcNAc blots were normalized to immunoprecipitated MAVS blots (E) Ubiquitination assay was conducted to compare the ubiquitination levels of wild-type with the Δ249–257 mutant MAVS in HEK293 cells. (F) MTT assay was performed to measure cell viability over time in response to SeV (100 HAU) infection in HEK293 and A549 cells. *n.s* indicated not statistically significant.
